# Supplementary material for: Chromothripsis during telomere crisis is independent of NHEJ, and consistent with a replicative origin
Source: Genome Res. 2019 May;29(5):737–49. doi: 10.1101/gr.240705.118 (PMC6499312; doi:10.1101/gr.240705.118)
Supplement: Supplemental Material [file supp_gr.240705.118_Supplemental_file_1.zip › contigs/annotated_contigs/DB47/contig.2.DB47_length_776_mean_cov_7.92525773196.docx]

**DB47_length_776_mean_cov_7.92525773196**

```
TGTGGGTGTTCTTTAAGGAAAAGCAAGTAAGACGAATATCGTACAACCCACCTAACTGCAATTGAATCAGTGGATGCTTCACTCGCCTG
 >chrX:10602748-10603201 + E=1e-260
GAATTCGTCTGTTCTTGGTGTGTTTAATAATGAGTCATGCGATACATTTGCAAGGCAATGGGGCCACCTCCCAGGGCCTGGGGCCACGT

TATGATTTTCTGCGCCTGAGTCCCCCGTCTTTGTTCAGACAGGTTGCTAATGCATCCCGCGAAGCTGACCTAATGCTACTTCAGTGGGT

CTATTTTTCTTCTGTGGCTCAGCTCGAATGTGATATTCAGGAAGTCGACTGTCAGGCATGCAAATTGAGAGAAAAATATGCCACATTAA

ATCATGATGGTTTAATAAGGTATTACTCATGGGCAAATGGCTTCTGGTACTCTCCTTAGTAGGGGCAAGTTGAAAAGCATAACCTGCCC

CAGA|GAAG|GAAAATTCAAAGCAATGGAAACAAAATCAGTCCTTACAGGTTTCACCGTTGGGATTTGGACCCGGAGTGAAAAGAGGAT
     >chrX:10604487-10604814 + E=7e-185
AATGGTCTCAGAGCAGCCTCTGGTGGCTGAATGTCAAAAGCTCAAGAAGTCAGAGGCCACCTTACTAGTGTTCTCTTGACCTCCACTCT

CCACGGTCTTAAGATTTTGTGCAAAGGAAGTTACTATTTAAATGACTTCAAGGCACTAGTTCAGAATTGATTAGGTTGGAATTGAAGTG

AATTCATCAGTTTCAAGGGCAATCACAGATGGACTATGGATTTGATGCTGACAAACTGGGCCATTC
```
